# MMP-9 overexpression is associated with intragenic hypermethylation of MMP9 gene in melanoma

**DOI:** 10.18632/aging.100951

**Published:** 2016-04-25

**Authors:** Luca Falzone, Rossella Salemi, Salvatore Travali, Aurora Scalisi, James A. McCubrey, Saverio Candido, Massimo Libra

**Affiliations:** ^1^ Department of Biomedical and Biotechnological Sciences, Laboratory of Translational Oncology & Functional Genomics, Section of General & Clinical Pathology and Oncology, University of Catania, 95124, Catania, Italy; ^2^ Oncological Pathology Unit, ASP, Catania, Italy; ^3^ Department of Microbiology and Immunology, Brody School of Medicine, East Carolina University, Greenville, NC 27858, USA

**Keywords:** MMP-9, epigenetic, intragenic, methylation, melanoma

## Abstract

Tumor spreading is associated with the degradation of extracellular matrix proteins, mediated by the overexpression of matrix metalloproteinase 9 (MMP-9). Although, such overexpression was linked to epigenetic promoter methylation, the role of intragenic methylation was not clarified yet. Melanoma was used as tumor model to investigate the relationship between the DNA intragenic methylation of *MMP9* gene and MMP-9 overexpression at transcriptional and protein levels. Computational analysis revealed DNA hypermethylation within the intragenic CpG-2 region of *MMP9* gene in melanoma samples with high MMP-9 transcript levels. In vitro validation showed that CpG-2 hotspot region was hypermethylated in the A375 melanoma cell line with highest mRNA and protein levels of MMP-9, while low methylation levels were observed in the MEWO cell line where MMP-9 was undetectable. Concordant results were demonstrated in both A2058 and M14 cell lines. This correlation may give further insights on the role of *MMP-9* upregulation in melanoma.

## INTRODUCTION

Metastatic spreading is the major cause of death in tumor patients [1,2]. Several molecules have been shown to contribute to tumor invasion and spreading. Among these, matrix metalloproteinase 9 (MMP-9) overexpression has been associated with tumor dissemination [3]. This evidence suggests its role as a prognostic factor.

However, the increased levels of MMP-9 have been described in several pathologic conditions and/or inflammatory status suggesting that it is not clearly specific in cancer [4]. Accordingly, several inflam-matory cytokines and growth factors that in turn may activate the Activator Protein-1/A Polyoma Enhancer Binding Protein-3 factor (AP-1/PEA3) and NF-kB led MMP-9 overexpression [5,6]. Constitutive increased expression of MMP-9 may be determined by genetic modifications such as polymorphisms. Among these, the C-1562T polymorphism, upstream to the start site of transcription, and the alteration of di-nucleotide CA repeats in the AP-1 regulatory sequence, were more frequent [7].

Further mechanisms of gene modulation have been associated with epigenetic modifications. In particular, the overexpression of different genes was related to the hypomethylation of gene promoter through chromatin decondensation, allowing the recruitment of the transcriptional complex. Moreover, demethylation of consensus sequence of transcription factors lets the binding of these transcription factors with promoter regions resulting in the gene upregulation, including that of *MMP9* [8,9,10].

Most recently, it was demonstrated that intragenic DNA methylation could affect the gene expression [10]. The authors reported that the intragenic methylation is positively correlated with the expression of the same gene and negatively correlated with the majority of histone modifications. In particular, it was speculated that intragenic methylation might have roles in the mechanisms of transcriptional elongation, intragenic activation (enhancer) and alternative splicing [10].

According to these evidences, the analysis of cancer epigenetic modifications is the best approach to discriminate if MMP-9 overexpression is directly associated with tumor progression and not with co-morbidities or inflammatory status. Therefore, in the present study we performed in silico and in vitro analyses to assess the methylation patterns of *MMP9* gene in melanoma and its implication in MMP-9 mRNA overexpression [11]. The choice of this tumor type was based on its highly invasive and metastatic characteristics where MMP-9 overexpression may play an important role [3].

## RESULTS

### Computational identification of an intragenic methylation hotspot in the *MMP9* gene

**Figure 1:**
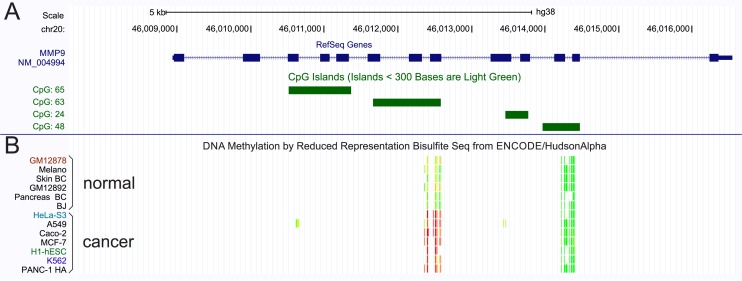
MMP9 methylation pattern (**A**) Computational detection of CpG islands within the MMP9 locus. (**B**) Methylation status of MMP9 gene, performed by ENCODE RRBS tool, in 6 normal cells compared to 7 cancer cells.

Four CpG islands were identified within the *MMP9* locus using the bioinformatic tool CpG Islands Tracks available in UCSC Genome browser (https://genome-euro.ucsc.edu). These regions were named CpG-1, CpG-2, CpG-3, CpG-4 according to their relative position in the *MMP9* sequence (Figure 1A, Table S1). In addition, the methylation analysis of *MMP9* gene was performed comparing 6 normal cell lines with 7 cancer cell lines using the bioinformatic ENCODE DNA Methylation RRBS (Figure 1B). As reported in the Figure 1B, the cancer cell lines showed a hypermethylated region (red and yellow bars) inside the annotated CpG-2 island compared to the normal cell lines (green bars). This region, located at chromosomal position CH20:46012338-46012584 (GRCh38 Primary Assembly), was reported in this study as a CpG-2 hotspot region.

### MMP-9 correlated levels of intragenic hypermethylation and mRNA expression in melanoma: computational analysis

**Figure 2:**
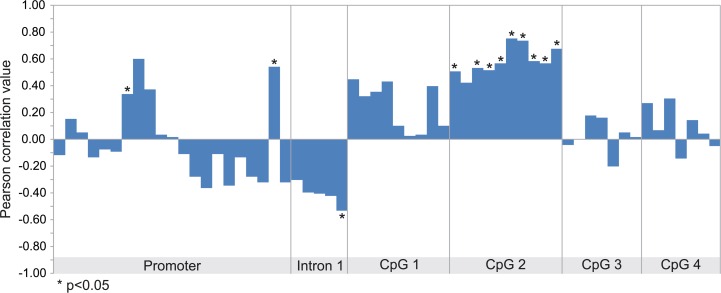
Correlation between MMP-9 expression and methylation status of MMP9 gene Pearson correlation analysis between methylation levels of each probeset and MMP-9 expression performed in all samples included in GSE31879 dataset.

**Figure 3:**
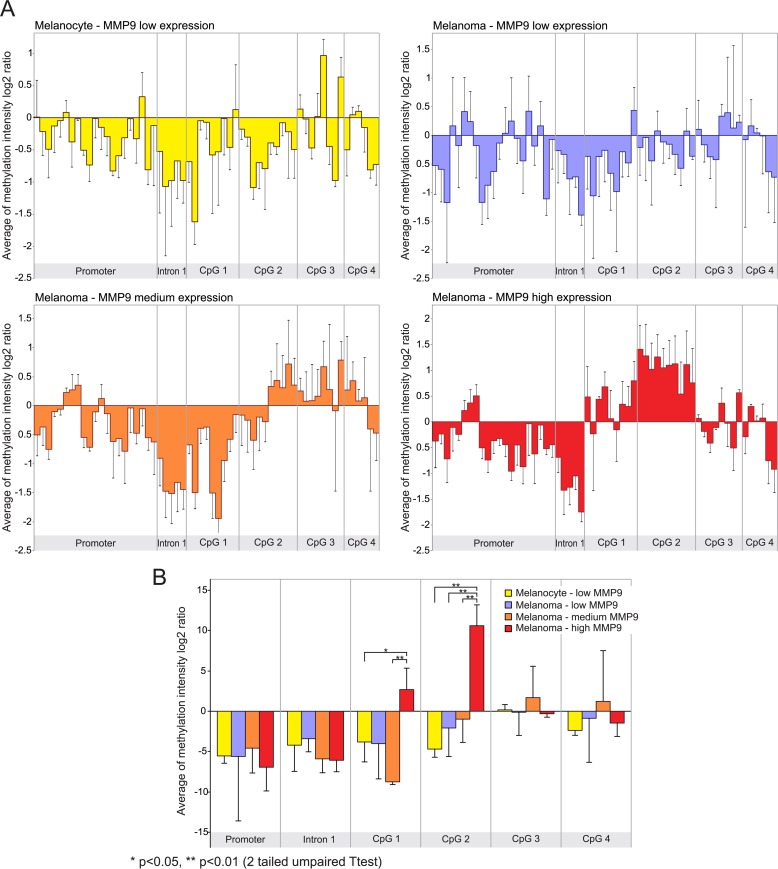
Differential analysis of MMP-9 expression and methylation status of MMP9 gene according to MMP-9 expression levels (**A**) Methylation analysis of MMP9 locus in the MMP-9 expression groups of melanoma samples, and melanocyte controls. (**B**) Cumulative statistical analysis of methylation levels of MMP9 in stratified expression groups.

The transcript levels of *MMP9* in the samples analyzed in GSE31879 melanoma dataset were about 1.5 fold higher in melanoma samples compared to melanocyte controls (p<0.01) (Figure S1A). Afterwards, we proceeded to stratify melanoma samples into low (below 30th percentile), medium (between 30th and 70th percentile) and high (above 70th percentile) groups according to the levels of MMP-9 mRNA expression. Statistical analysis revealed that in the high and medium groups, MMP-9 levels were significantly overexpressed as compared to both the low group and melanocytes (p<0.01) (Figure S1B). Furthermore, Pearson correlation analysis was performed between MMP-9 expression levels and methylation intensity of *MMP9*-specific probes in selected samples included in GSE31879 dataset. The statistical analysis reveals a moderate positive correlation (p<0.05) between MMP-9 levels and methylation status of probes belonging to CpG-2 group (Figure 2, Table S2). When the melanoma samples were stratified in high, moderate and low group according the levels of MMP-9 expression, the CpG-2 region was hypermethylated in the MMP-9 high-expression melanoma samples (box 4) compared to other groups, including melanocyte controls (box 1-3) (Figure 3A). The cumulative statistical analysis of methylation levels of *MMP9* showed a significant difference among the four groups in CpG-2 region (p<0.01); while, in GpG-1 island statistical significance difference was observed only for high vs medium (p<0.01) and high vs low (p<0.05) (Figure 3B).

### Positive correlation between MMP-9 expression and hyper-methylation of CpG-2 hotspot in melanoma cell lines

**Figure 4:**
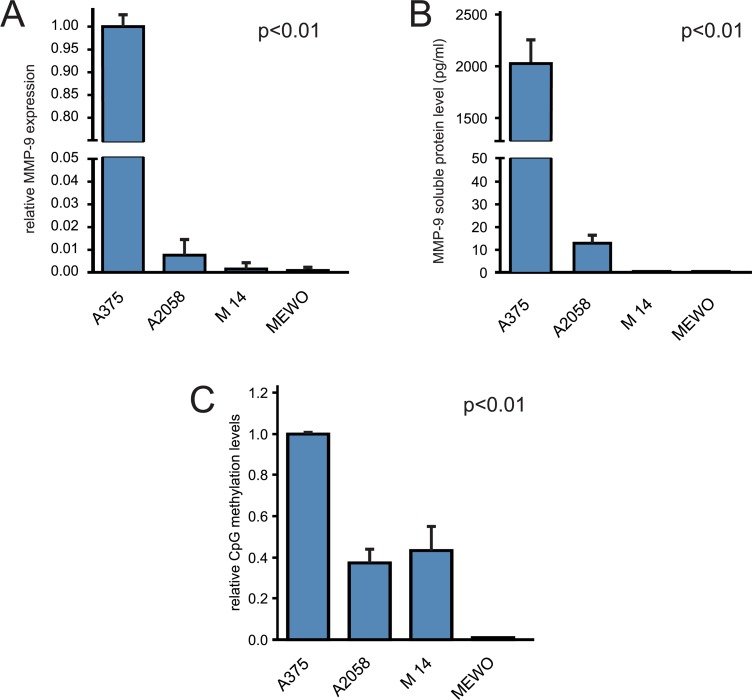
Correlation of MMP-9 CpG-2 hotspot with MMP-9 expression in melanoma cell lines (**A**) RT-qPCR analysis of MMP-9 transcription levels. (**B**) ELISA determination of released MMP-9 in cell medium supernatant. (**C**) Methylation analysis of MMP9 CpG-2 hotspot in melanoma cell lines by MSRE assay. One-way ANOVA test was performed to analyze statistical significance.

Protein and mRNA levels of MMP-9 were tested in A375, A2058, M14 and MEWO melanoma cell lines by ELISA test and RT-qPCR, respectively. Real-time analysis revealed that *MMP-9* gene expression was 100-fold higher in A375 compared to other cell lines (Figure 4A). Similar results were obtained by ELISA. Soluble MMP-9 levels were 2024.6 pg/mL for A375 and 13.2 pg/mL for A2580, whereas M14 and MEWO cell lines showed MMP-9 protein levels undetectable by the ELISA kit used in this study (Figure 4B).

Methylation status of *MMP9* at CpG-2 hotspot sequence, as identified by computational approach, was analyzed using the methylation-specific restriction enzyme (MSRE) assay. This was performed as a one-step protocol using the methylation-sensitive HpaII restriction enzyme, that is able to digest the unmethylated DNA but not methylated at 5′-CCGG-3′sites. Digested DNA and non-digested DNA (control reference), of each melanoma cell line samples were subjected to q-PCR to amplify the putative CpG-2 hotspot region, containing 6 HpaII consensus sites.

As expected, higher amplification levels of CpG-2 hotspot sequence were observed in A375 compared to other cell lines. A2058 and M14 showed lower relative methylation levels (< 50%) compared to those observed in A375. While, no amplification signal was observed in the MEWO cell line (Figure 4C).

## DISCUSSION

Despite recent advances in therapy of metastatic melanoma, the survival rate of patients with melanoma is still low [12,13,14]. The identification of specific prognostic markers suitable to recognize aggressive phenotype in melanoma may improve patient clinical outcome [15].

To our best knowledge, this is the first report describing the intragenic methylation as an additional potential molecular mechanism responsible for the *MMP9* upregulation in cancer. The degradation of pericellular and stromal compartments, mainly mediated by MMP-9, is an essential process during melanoma invasion and migration [3,16]. *MMP9* transcription is strictly regulated by several cytokines and cell/cell or cell/matrix interactions, furthermore the cleavage of proMMP-9 is required for the full proteolytic activity [5,17]. The activation of MMP-9 in cancer has been associated with tumor growth and tumor spreading [18]. Notably, Rangaswami [19] described that MMP-9 is activated in melanoma [19]. The abnormal expression of MMP-9, occurring in cancer cells, may be sustained by hypomethylation or genomic alterations of its promoter [7,11]. All these observations suggested the rational to study MMP-9 in cancer.

Here, we proposed that the intragenic DNA methylation could affect MMP-9 expression. This hypothesis was supported by Singer *et al*. [10]. The authors showed that the intragenic methylation was positively correlated to the gene expression and negatively correlated with the majority of histone modifications involved in gene silencing. Mechanically, the intragenic methylation may affect the transcription elongation, intragenic activation (enhancer) and alternative splicing of several genes [10].

On these bases, we first performed computational analysis to assess the methylation patterns of *MMP9* gene in melanoma samples according to mRNA expression. Such analysis revealed that hypermethylation was observed at the CpG-2 intragenic region in the group with higher MMP-9 expression levels. Accordingly, our in vitro experiments demonstrated that MMP9 CpG-2 methylation hotspot was correlated with higher transcript and protein levels of MMP-9 in melanoma cell lines. Then, the intervention of epigenetic modifications may be associated with MMP-9 overexpression.

Our results further underline that tumor DNA appears to be the best source to identify the molecular signature of tumor for each patient. The recent advances in the field of biotechnology allow the use of the circulating tumor DNA for the identification of molecular abnormalities, giving a dynamic view of such molecular modifications to evaluate treatment efficacy and patient follow-up [20,21,22]. Among these abnormalities, we might include the *MMP9* intragenic methylation hotspot that is responsible of the MMP-9 overexpression.

Overall, the results of the present study support the notion that *MMP9* intragenic hypermethylation is associated with MMP-9 overexpression that in turn plays a role in cancer development and progression. Further-more, our data are in agreement with previous studies in which DNA methylation were identified as prognostic markers in cancer [23,24,25,26]. Finally, the increased levels of MMP-9, when associated with such intragenic methylation hotspot, may predict a malignant phenotype.

## MATERIALS AND METHODS

### Computational identification of CpG islands and methylation status of *MMP9* gene

The CpG islands of *MMP9* gene were identified by computational approaches available on UCSC Genome browser (https://genome.ucsc.edu) (Table S1 and Figure 1A). This tool shows the genomic region satisfying the following conditions: CG content greater than or equal to 50%, length greater than 200 bp and ratio is greater than 0.6 of observed number of CG dinucleotides to the expected number on the basis of the number of Gs and Cs in the segment [27].

Moreover, computational analysis of CpG methylation of *MMP9* locus were explored using ENCODE DNA Methylation by Reduced Representation Bisulfite Sequencing (RRBS) Tracks developed by Encyclopedia of DNA Elements, available on UCSC Genome browser (Figure 1B). This track reports the percentage of DNA fragments that exhibit methylation at specific CpG dinucleotides. DNA methylation status was assayed using RRBS. Briefly, Genomic DNA, extracted from several cell lines, was digested with the methyl-insensitive restriction enzyme MspI. After purification, the small genomic DNA fragments were used to construct an Illumina sequencing library. This genomic library was treated with sodium bisulfite and amplified by PCR to convert every unmethylated cytosine in a thymidine while methylated cytosines were protected from bisulfite conversion. The sequenced fragments were aligned to the reference genome sequence to calculate the percentage of sequence that showed methylated CpG dinucleotides [28].

### Melanoma dataset analysis for MMP-9 mRNA expression and methylation

Publicly available Gene Expression Omnibus (GEO) datasets of melanoma were analyzed to evaluate the association between MMP-9 mRNA expression and methylation status of *MMP9* locus. Only datasets with both gene expression and DNA methylation profiling by genome tiling array were considered for the analysis. According to these criteria only the dataset GSE31879 was suitable for the present study. Expression data were obtained using Affymetrix Human Genome U133 Plus 2.0 while methylation profiling data were evaluated by Human DNA Methylation 3x720K CpG Island Plus RefSeq Promoter Array platform (Roche NimbleGen, Inc. - Germany).

In this dataset only 10 of 11 melanoma specimens and 3 of 5 melanocyte cells samples exhibited both mRNA expression levels and DNA methylation status. Melanoma samples were stratified into 3 groups: low (below 30th percentile), medium (between 30th and 70th percentile) and high (above 70th percentile) according to MMP-9 mRNA expression (Figure S1). Methylation-sensitivity probes specific for *MMP9* gene, were grouped into Promoter, Intron-1, CpG-1, CpG-2, CpG-3 and CpG-4 regions according to alignment position of each probe with the promoter, the first intron and the previously named CpG islands of *MMP9* gene (Table S2, Figure 2 and 3).

### Cell lines and culture conditions

Melanoma cell lines A375, A2058 and MEWO were obtained from the ATCC (Rockville, MD, USA). M14 cell line was available at the Department of Biomedical and Biotechnological Sciences of the University of Catania.

Cells were maintained in a humidified 5% CO_2_ incubator at 37°C with RPMI-1640 for A375, A2058 and M14 while EMEM was used for MEWO. Both media were supplemented with 2 mmol/L L-glutamine, 100 IU penicillin and 100 μg/ml streptomycin and 10% fetal bovine serum (FBS). All media and supplements were provided from Lonza (Walkersville, USA).

All cell lines were plated in triplicate into 100 mm cell-culture dishes (Thermo Fisher Scientific Inc., USA) and grown under normal culture conditions to 80% confluency. Supernatants of each cell culture were harvested and centrifugated to remove cellular debris. Adherent cells were washed once and scraped in 1X DPBS (Lonza, Walkersville, USA). Cellular pellets were collected via centrifugation and stored at −80°C.

### RT-qPCR analysis

Total cellular RNAs were extracted from cultured cell lines with The PureLink® RNA Mini Kit (Thermo Fisher Scientific Inc., USA) according to the manufacturer's instructions. The concentration and purity of the RNAs were ascertained on a NanoDrop spectrophotometer (Thermo Scientific). Reverse transcription was carried out using M-MLV reverse transcriptase (Invitrogen) and random primers (Invitrogen). SYBR green-based real time PCR was conducted with the Applied Biosystem 7500 Real-Time PCR System using SYBR Green PCR Master Mix (Applied Biosystem, USA). The amplification of *MMP9* and the Phosphoglycerate Kinase 1 (*PGK1*) cDNAs, were performed in triplicate using primers and amplification conditions reported in Table S3. The ddCt relative quantification method was performed to quantify the expression of MMP-9 using PGK-1 signal value as control reference.

### ELISA

MMP-9 protein levels were detected in supernatants of each melanoma cell culture using MMP-9 Human ELISA Kit (Invitrogen) according to the datasheet. Briefly, the plate was coated in duplicate with 100 μL of supernatants, standards and controls. The plate was left at RT for 2 hours. After washing four times with wash buffer, 100 μL of streptoavidin-HRP conjugated were added to plate and incubated for 30 min at RT. Then, the plate was washed four times and 100 μL of stabilized chromogen were added to each well. After 30 min incubation, the substrate reaction was stopped with stop solution. Finally, optical density (OD) was measured by Tecan ELISA plate reader (Tecan, Switzerland). The averages of duplicate readings of standards and controls were used to generate the standard curve by linear regression analysis. MMP-9 concentrations were calculated fitting the average of duplicate ODs of each sample with standard curve.

### Methylation-specific restriction enzyme assay (MSRE)

Methylation-specific restriction enzyme (MSRE) assay is based on use of endonucleases that are not able to cleave methylated-cytosine residues contained in specific consensus sites, leaving methylated DNA intact. Amplification levels of target sequence depend on its content of methylated CpGs, in particular low amplification signal is observed in hypomethylated DNA while higher signal is detected in hypermethylated sequence.

Genomic DNA was extracted from melanoma cell lines using Illustra triplePrep Kit (GE Healthcare, USA) according to manufacturer's instructions. DNA of each sample was digested with restriction enzymes HpaII (New England Biolabs) as explained below.

Each restriction mixture, equally distributed in 0.2 ml reaction tubes, was composed of 50 units of HpaII (5 μL), 5 μL of corresponding 10X REact 8 (New England Biolabs), 1 μg of DNA sample and filled to 50 μL with DNAse free water. Similarly, for each sample the same restriction mixtures without HpaII enzyme were prepared and used as control reference. The methylation insensitive MspI restriction enzyme was used to test the efficiency of MSRE assay.

The restriction reactions and matching controls were incubated at 37°C for 8 hours. Subsequently, 2 μL of Proteinase K (NEB) (20 mg/mL) were added to each tube and placed at 40°C for 30 min, followed by denaturation at 95°C for 10 min. 5 μL of all samples (digested DNA and controls) were subjected to real SYBR green-based real time PCR. The CpG-2 hotspot region, as identified by bioinformatic approaches, was amplified using primers and amplification conditions as shown in table S3. All analyses were performed in triplicate.

### Statistics analysis

No additional normalization procedures were applied to data obtained from GSE31879 dataset included in this study. For each MMP-9 expression group, above described, we performed the average of cumulative methylation probe values of the promoter region, of the intron 1 element, and the annotated CpG islands, previously identified in *MMP9* locus. Student's t-Test, one-way ANOVA test and Pearson correlation analysis were performed by R software (www.r-project.org).

## SUPPLEMENTARY DATA


