# Supplementary material for: MMP-9 overexpression is associated with intragenic hypermethylation of MMP9 gene in melanoma
Source: Aging (Albany NY). 2016 Apr 25;8(5):933–40. doi: 10.18632/aging.100951 (PMC4931845; doi:10.18632/aging.100951)
Supplement: Supplementary file 1 [file aging-08-0933-s001.pdf]

SUPPLEMENTARY DATA

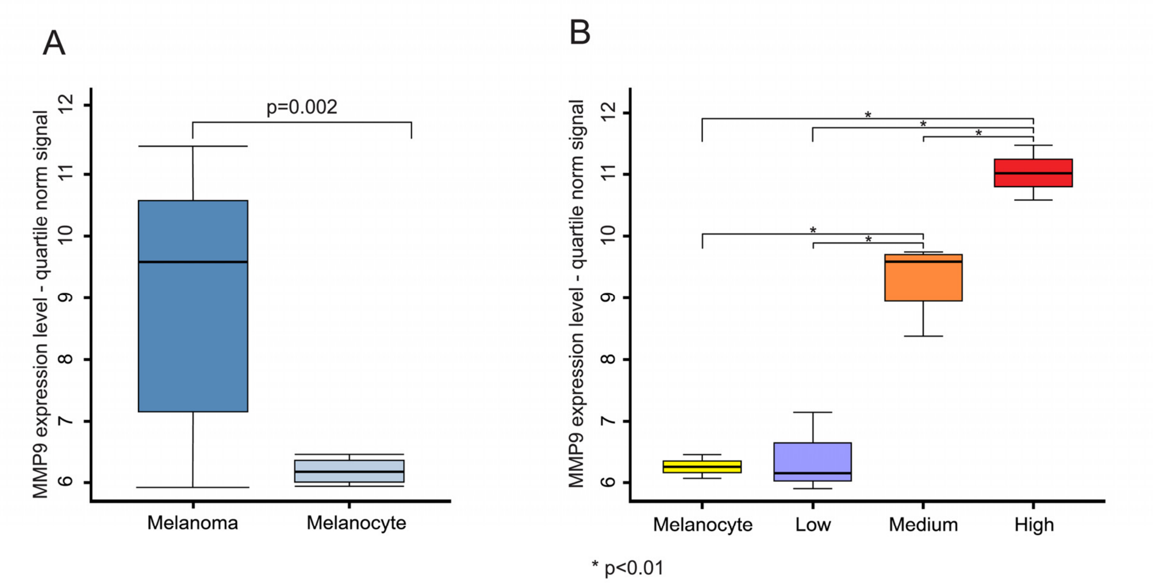

Figure S1. Microarray analysis of MMP-9 expression levels in melanoma samples. (**A**) Differential analysis of MMP-9 expression between melanoma samples and melanocyte controls. (**B**) Statistical analysis among melanoma samples stratified in low, medium and high expression group according the MMP-9 expression levels.

Table S1. CpG islands of MMP-9 gene according to CpG Islands Tracks available in UCSC Genome browser.

Region name	*Chromosome position	Size	CpG count
CpG 1	chr20:46010497-46011349	853	65
CpG 2	chr20:46011650-46012571	922	63
CpG 3	chr20:46013456-46013767	312	24
CpG 4	chr20:46013967-46014472	506	57

*Human Dec. 2013 (GRCh38/hg38) Assembly

Table S2. Pearson Correlation analysis between methylation probset levels and the levels of MMP-9 expression. For each probset, chromosome position, nucleotide sequence and region in which it belongs were reported.

Region	*Chromosome position	Sequence	R	p
Promoter	chr20:46006492-46006543	TCATATTACTCTATGACCTCACATTCCCG AGTCTAGAATCTAGTTCCTCCTG	-0.14	0.5549
Promoter	chr20:46006592-46006641	TTACCTTCAGTGGGTAGCAGGGGACCAG AGGAGAAGCATCCAGGTTTTAT	0.18	0.8355
Promoter	chr20:46006668-46006724	TACCCACTTCTATACCTGGGTCATCACA GTTCCCTGTAAATGGTAATAAAGATGAA A	0.06	0.5903
Promoter	chr20:46006768-46006817	GGATTAACTCGCTCTGTGATCACAGGCA AATTCCTTAACTCTCTGAGCCT	-0.16	0.7741
Promoter	chr20:46006870-46006920	TGGTGAGGATGAAACGAGAGGCTTATAG AGAACTTATTACGGTGCTTGACA	-0.09	0.7255
Promoter	chr20:46006992-46007041	CTGGAAAATGGCAGAGCCGGGATGGAA ATCCAGGACTTCGTGACTGCAAA	-0.11	0.1782
Promoter	chr20:46007090-46007152	AGGAAGTTAATTATCTCCATCTCACAGT CTCATTTATTAGATAAGCATATAAAATG CCTGGCA	0.4	0.0063
Promoter	chr20:46007492-46007542	CAACTTTTTGAGTTGTTAGCAGGTTTTTC CCAAATAGGGCTTTGAAGAAGG	0.71	0.1293
Promoter	chr20:46007592-46007641	GGAGGCTGCTGGTGTGGGAGGCTTGGGA GGGAGGCTTGGCATAAGTGTGA	0.44	0.8936
Promoter	chr20:46007674-46007723	AGGGCTGGAGAACTGAAAGGGCTCCTAT AGATTATTTTCCCCCATATCCT	0.04	0.956
Promoter	chr20:46007788-46007838	TGCAGCTTAGAGCCCAATAACCTGGTTT GGTGATTCCAAGTTAGAATCATG	0.02	0.956
Promoter	chr20:46007992-46008041	GCCCTGAATCTTGGGTCTTGGCCTTAGTA ATTAAAACCAATCACCACCAT	-0.13	0.6784
Promoter	chr20:46008096-46008145	TTTAATCCTCACATCAATTTAGGGACAA AGAGCCCCCCACCCCCCGTTTT	-0.33	0.2704
Promoter	chr20:46008202-46008251	AAGGAAGAGAGTAAAGCCATGTCTGCTG TTTTCTAGAGGCTGCTACTGTC	-0.43	0.1433
Promoter	chr20:46008314-46008363	AGCCTTGCCTAGCAGAGCCCATTCCTTC CGCCCCCAGATGAAGCAGGGAG	-0.13	0.6831
Promoter	chr20:46008398-46008447	AAAAAGAGGACAGAGCCTGGAGTGTGG GGAGGGGTTTGGGGAGGATATCT	-0.41	0.1593
Promoter	chr20:46008502-46008551	TTCAGAAAGAAGTCTCAGGGAGTCTTCC ATCACTTTCCCTTGGCTGACCA	-0.16	0.6093
Promoter	chr20:46008602-46008651	TCCCTCCCTTTCATACAGTTCCCACAAGC TCTGCAGTTTGCAAAACCCTA	-0.33	0.2647
Promoter	chr20:46008692-46008741	TCTTGCCTGACTTGGCAGTGGAGACTGC GGGCAGTGGAGAGAGGAGGAGG	-0.38	0.1972
Promoter	chr20:46008810-46008859	ACACACACACCCTGACCCCTGAGTCAGC ACTTGCCTGTCAAGGAGGGGTG	0.64	0.0187
Promoter	chr20:46008892-46008941	AACAGCAGCTGCAGTCAGACACCTCTGC CCTCACCATGAGCCTCTGGCAG	-0.38	0.1987
Intron 1	chr20:46009008-46009057	CTTGTGCTCTTCCCTGGAGACCTGAGAA CCAATCTCACCGACAGGCAGCT	-0.36	0.2273
Intron 2	chr20:46009108-46009157	GGGTGTTGAGTGTCCCAGAGAGGATGCA GGGCCTCAGAGGAGATGCTTTA	-0.47	0.1054
Intron 3	chr20:46009208-46009257	TTAGGCAGTGGGGGGTCTTGTGGAGGCT TTGAGCAGTGATGGCCAGAAAT	-0.48	0.1008

Intron 4	chr20:46009317-46009366	GAGGGTTCTGGGGTAAGCATAGGCTGGG AGTGAACAGGGGCAAACCTTAT	-0.5	0.0813
Intron 5	chr20:46009413-46009462	GAGCTGAGGATGTCTAAGGAGGGGAGA TCCCTGGGTGGTCAGAAAGCACT	-0.63	0.0216
1CpG	chr20:46010496-46010545	TCGGAAGACTTGCCGCGGCGGTGATTG ACGACGCCTTTGCCCGCGCCTT	0.53	0.0601
1°CpG	chr20:46010604-46010653	GACATCGTCATCCAGTTTGGTGTCGCGG GTGAGAACGTGAGGAGGGAAAA	0.38	0.195
1°CpG	chr20:46010720-46010769	GGCTTCCTCTTGCCTGCCCGCGCTGCCCT GGCTTATACGGCCCCTCCTGC	0.42	0.1579
1°CpG	chr20:46010804-46010853	AGAGCTTCGCGCAGGCGGGATTTCAGCC CGCACTTATTTCGGAGCCCTTG	0.51	0.078
1°CpG	chr20:46010912-46010961	GTTTCTTCAGAGCACGGAGACGGGTATC CCTTCGACGGGAAGGACGGGCT	0.12	0.7051
1°CpG	chr20:46011008-46011057	CATTTCGACGATGACGAGTTGTGGTCCC TGGGCAAGGGCGTCGGTGAGAT	0.03	0.9121
1°CpG	chr20:46011116-46011165	TAACTCCGGTCCCCCCTCCTCCTGCAGTG GTTCCAACTCGGTTTGGAAAC	0.04	0.8886
1°CpG	chr20:46011216-46011265	CTCTGCCTGCACCACCGACGGTCGCTCC GACGGCTTGCCCTGGTGCAGTA	0.47	0.1047
1°CpG	chr20:46011296-46011345	TTGGCTTCTGCCCCAGCGAGAGTGAGTG AGGGGGCTCGCCGAGGGCTGGG	0.12	0.6945
2°CpG	chr20:46011672-46011721	TCCGACGGCTACCGCTGGTGCGCCACCA CCGCCAACTACGACCGGGACAA	0.6	0.0313
2°CpG	chr20:46011750-46011799	ACCTCCACCCTGTCTACCAGGTTCAGCC CCGCCCTCTCATCATGTATTGG	0.5	0.0789
2°CpG	chr20:46011868-46011917	TGACTCCGCCCACCTACACCACATTTCC ACCACTATCCCTGACTTCCAAT	0.63	0.0215
2°CpG	chr20:46011968-46012017	TCTTCCTTGGTCTGGTGTCCCAGGCACCG CCCACGGGTCTAGCCTCTTCT	0.61	0.0265
2°CpG	chr20:46012056-46012105	GTTTAGCTCCCTGTCGGGTCGGCCCCTG ACTCCTTATTGGACTCATCCAT	0.67	0.0114
2°CpG	chr20:46012174-46012223	GCTGTGCGTCTTCCCCTTCACTTTCCTGG GTAAGGAGTACTCGACCTGTA	0.89	0
2°CpG	chr20:46012250-46012299	CTCTGGTGCGCTACCACCTCGAACTTTG ACAGCGACAAGAAGTGGGGCTT	0.87	0.0001
2°CpG	chr20:46012374-46012423	CAGGGCTGGGGGCTCGGCCCGGCGCTCA CGTCTCAGGCTCCCTCTCCCTC	0.69	0.0089
2°CpG	chr20:46012472-46012521	TGGGCTTAGATCATTCCTCAGTGCCGGA GGCGCTCATGTACCCTATGTAC	0.67	0.0127
2°CpG	chr20:46012550-46012599	AGGACGACGTGAATGGCATCCGGCACCT CTATGGTGAGGCAGGGGCAGGG	0.8	0.0009
3°CpG	chr20:46013265-46013314	TGAACCTGAGCCACGGCCTCCAACCACC ACCACACCGCAGCCCACGGCTC	-0.05	0.8632
3°CpG	chr20:46013379-46013428	CACAGGTCCCCCCTCAGCTGGCCCCACA GGTCCCCCCACTGCTGGCCCTT	0	0.9947
3°CpG	chr20:46013461-46013510	GACGATGCCTGCAACGTGAACATCTTCG ACGCCATCGCGGAGATTGGGAA	0.21	0.5001
3°CpG	chr20:46013585-46013634	GCCCGTCCCTTCCCGCCCACTGGCCCTGT GTCCAAGGCTTAGAGCCCGTC	0.19	0.5286
3°CpG	chr20:46013685-46013734	GGGAGCCGGCCGCAGGGCCCCTTCCTTA TCGCCGACAAGTGGCCCGCGCT	-0.24	0.4315
3°CpG	chr20:46013763-46013812	GAGCGGCTCTCCAAGAAGCTTTTCTTCTT CTCTGGTTAGTTACCTACTTT	0.06	0.8503
3°CpG	chr20:46013862-46013911	ATCGATAACCCACGAAACGTCTTGTGCG TTTTAGAAAAATACGCCCCCTG	0.02	0.9419

4°CpG	chr20:46013962-46014011	CTCCACGCCCTCGCGTCGCTCTACCCAG CGCCTCTGCCCCTGGGTTGCAG	0.32	0.2922
4°CpG	chr20:46014066-46014115	TCTAGGAGTACGTGCTCCCTCTGCGCCC CCAAACCGACGTGACCCTCCTC	0.08	0.8071
4°CpG	chr20:46014164-46014213	CCCGAGGCGTCTGGACAAGCTGGGCCTG GGAGCCGACGTGGCCCAGGTGA	0.36	0.2217
4°CpG	chr20:46014286-46014335	CGCGGCCGCCGGCAGGGGGAGCCCGGG CGCCGTCGGTCCGTCCGCTAGCC	-0.17	0.5722
4°CpG	chr20:46014372-46014421	TTCGACGTGAAGGCGCAGATGGTGGATC CCCGGAGCGCCAGCGAGGTGGA	0.17	0.5791
4°CpG	chr20:46014482-46014531	CTGAGGAGGATCCCTTCGTGAGACACCA CACTAAGCTCCTCTTAGTGAGT	0.05	0.8791
4°CpG	chr20:46014586-46014635	AGCACAGACAAGATCCCAGCAGAGGCA GAGGCCTTCTCCAGGTCATTTAG	-0.06	0.836

*Human Dec. 2013 (GRCh38/hg38) Assembly

Table S3. Primers and real time amplification conditions.

RT-qPCR:		
MMP-9 forward	5'-GAACCAATCTCACCGACAGG-3'	94°C for 10 min, followed by forty cycles of 94°C for 15 s, 64°C for 40 s and 72°C for 1 min.
MMP-9 reverse	5'-CCACAACTCGTCATCGTCG-3'	
PGK-1 forward	5'-TTAAAGGGAAGCGGGTCGTT-3'	
PGK-1 reverse	5'-CAGGCATGGGCACACCAT-3'	
MSRE-qPCR:		
CpG 2 hotspot forward	5'- GTGCGCTACCACCTCGAACT-3'	94°C for 10 min, followed by forty cycles of 94°C for 15 s, 62°C for 20 s and 72°C for 40 s.
CpG 2 hotspot reverse	5'-AGGCTCTGCTTCCAGACAGACG-3'	
